# Pyroptosis is a drug target for prevention of adverse cardiac remodeling: The crosstalk between pyroptosis, apoptosis, and autophagy

**DOI:** 10.7555/JBR.36.20220123

**Published:** 2022-08-10

**Authors:** Natalia V. Naryzhnaya, Leonid N. Maslov, Sergey V. Popov, Alexandr V. Mukhomezyanov, Vyacheslav V. Ryabov, Boris K. Kurbatov, Alexandra E. Gombozhapova, Nirmal Singh, Feng Fu, Jian-Ming Pei, Sergey V. Logvinov

**Affiliations:** 1 Laboratory of Experimental Cardiology, Cardiology Research Institute, Tomsk National Research Medical Center of the Russian Academy of Sciences, Tomsk, Tomsk Region 634012, Russia; 2 Department of Pharmaceutical Sciences and Drug Research, Punjabi University, Patiala 147002, India; 3 Department of Physiology and Pathophysiology, National Key Discipline of Cell Biology, School of Basic Medicine, Fourth Military Medical University, Xi'an, Shaanxi 710032, China; 4 Department of Histology, Embryology and Cytology, Siberian State Medical University, Tomsk, Tomsk Region 634055, Russia

**Keywords:** pyroptosis, apoptosis, autophagy, cardiac remodeling, diabetes, metabolic syndrome

## Abstract

Acute myocardial infarction (AMI) is one of the main reasons of cardiovascular disease-related death. The introduction of percutaneous coronary intervention to clinical practice dramatically decreased the mortality rate in AMI. Adverse cardiac remodeling is a serious problem in cardiology. An increase in the effectiveness of AMI treatment and prevention of adverse cardiac remodeling is difficult to achieve without understanding the mechanisms of reperfusion cardiac injury and cardiac remodeling. Inhibition of pyroptosis prevents the development of postinfarction and pressure overload-induced cardiac remodeling, and mitigates cardiomyopathy induced by diabetes and metabolic syndrome. Therefore, it is reasonable to hypothesize that the pyroptosis inhibitors may find a role in clinical practice for treatment of AMI and prevention of cardiac remodeling, diabetes and metabolic syndrome-triggered cardiomyopathy. It was demonstrated that pyroptosis interacts closely with apoptosis and autophagy. Pyroptosis could be inhibited by nucleotide-binding oligomerization domain-like receptor with a pyrin domain 3 inhibitors, caspase-1 inhibitors, microRNA, angiotensin-converting enzyme inhibitors, angiotensin Ⅱ receptor blockers, and traditional Chinese herbal medicines.

## Introduction

Acute myocardial infarction (AMI) is one of the main causes of death and disability worldwide^[1]^. For patients suffering ST-elevation myocardial infarction (STEMI), minimizing ischemic time by urgent revascularization, either by way of percutaneous coronary intervention or thrombolytic therapy, has been a successful strategy in reducing mortality^[1]^. However, restoration of coronary flow may promote further myocardial injury through a process known as reperfusion injury^[2]^.

Adverse pathological remodeling of the heart is a process that is associated with cardiac fibrosis and heart failure^[3]^. Pathological hypertrophy is a process associated with myocardial fibrosis and capillary rarefaction, and is the result of imbalanced cardiac growth (cardiomyocytes and capillaries)^[3]^. Preventing postinfarction cardiac remodeling is as important as protecting the heart from reperfusion injury. Left ventricular remodeling is associated with the progression of postinfarction heart failure^[4]^. Adverse cardiac remodeling was developed in 11% of patients 8 years after myocardial infarction, and such patients are more likely in need of admission^[5]^. According to another data, left ventricular dilation was developed in 49% of subjects 12 months after STEMI^[6]^. It is believed that inflammation plays an important role in the pathogenesis of postinfarction cardiac remodeling^[7–8]^. Pyroptosis is one of the manifestations of the inflammatory response to ischemia/reperfusion (I/R) of the heart^[9]^. We believe that pyroptosis plays an important role in the development of adverse postinfarction cardiac remodeling. Pyroptosis is a programmed form of cell death closely associated with inflammation. It is accompanied by stimulation of toll-like receptors, nucleotide-binding oligomerization domain-like receptor with a pyrin domain 3 (NLRP3) formation, activation of caspase-1, interleukin-1β (IL-1β) and IL-18 formation and secretion, a transformation of gasdermin D to N-gasdermin D^[10]^.

NLRP3 plays a key role in pyroptosis. It is a molecular sensor that detects microbial motifs and endogenous danger signals resulting in the formation and activation of the NLRP3 inflammasome^[11]^. In 2002, the term "inflammasome" was first put forward by Martinon *et* *al*^[12]^. They labeled it as "a caspase-activating complex" which consists of pro-caspase-1, pro-caspase-5, apoptosis speck-like protein (ASC), and pyrin domain-containing protein. In their opinion, inflammasome catalyzes the conversion of pro-caspase-1 to caspase-1^[12]^. It is now generally accepted that the NLRP3 inflammasome is a cytoplasmic multiprotein complex consisting of NLRP3, ASC, and pro-caspase-1^[13–14]^. It is believed that pyroptosis begins with NLRP3 synthesis, which occurs under the impact of danger-associated molecular patterns (DAMPs) and pathogen-associated molecular patterns (PAMPs) that activate pattern recognition receptors (PRRs)^[15]^. PRRs are toll-like receptors (TLRs), primarily TLR4, interleukin-1 receptor (IL-1R), and tumor necrosis factor (TNF) receptor (TNFR)^[15–16].^ Stimulation of PRRs promotes the activation of nuclear factor κB (NF-κB) which induces NLRP3 and pro-interleukin-1β synthesis^[14]^.

In 2015, gasdermin D protein was discovered to be another crucial component of pyroptosis^[17–18]^. It demonstrated the presence of gasdermin D in the structure of the NLRP3 inflammasome^[17]^. Deletion of the gene encoding gasdermin D showed that gasdermin D was necessary for pyroptosis and IL-1β secretion^[17]^. Gasdermin D-deficient cells are resistant to pyroptosis induction^[18]^. Gasdermin D is cleaved and activated by caspase-1, leading to the formation of membrane pores, cell swelling, and rapid lysis, and the release of inflammatory cytokines IL-1β, and IL-18^[18–19]^. Consequently, pyroptosis may be defined as gasdermin-mediated programmed necrosis.

We have previously reported that pyroptosis plays an important role in ischemic/reperfusion cardiac injury^[10]^. We discussed the role of pyroptosis in adverse cardiac remodeling and the crosstalk between pyroptosis, apoptosis and autophagy in this review. We used PubMed as the main source of information and we obtained full-text articles from web sites of related journals.

## The role of pyroptosis in adverse cardiac remodeling

### The role of pyroptosis in postinfarction cardiac remodeling

In 2011, it was found that the activation of pyroptosis is continued at least within seven days after permanent coronary artery occlusion (CAO) in mice^[20]^. Consequently, it is reasonable to believe that pyroptosis may be involved in postinfarction cardiac remodeling. IL-1β is a key product of pyroptosis. Monoclonal IgG2a antibody against IL-1β was given immediately after permanent CAO in mice and repeated one week later^[9]^. Treatment with an IL-1β-antibody did not affect inflammasome formation in myocardial tissue at 72 hours after CAO but inhibited cardiomyocyte apoptosis, limited left ventricular enlargement and improved systolic and diastolic cardiac function 7, 28 and 70 days after myocardial infarction. However, an IL-1β-antibody had no significant effect on improving survival of mice after myocardial infarction. Rats were subjected to permanent CAO in another study^[21]^. The cathepsin B inhibitor CA-074Me was injected daily for four weeks. CA-074Me inhibited NLRP3, IL-1β, IL-18, and caspase-1 expression in myocardial tissue, and limited contractile dysfunction of the heart and hypertrophy of cardiomyocytes^[21]^. In 2015, a study showed that the activation of the NLRP3 inflammasome in M1 macrophages promoted cardiac fibrosis in rats with permanent CAO^[22]^. It was found that chronic administration of the NLRP3-inflammasome inhibitor MCC950 prevents the development of cardiac fibrosis in mice five weeks after permanent CAO^[23]^. According to a study that the mice with permanent CAO^[24]^, MCC950 was injected at a dose of 10 mg/kg intraperitoneally three times per week for two weeks. Permanent CAO resulted in heart failure, induced cardiac fibrosis and an increase in collagen content in myocardial tissue. Chronic administration of MCC950 significantly mitigates these alterations but not completely abolishes adverse remodeling of the heart.

In a study discussing the role of Dectin-1, a c-type lectin, in postinfarction cardiac remodeling, mice were subjected to permanent coronary artery ligation^[25]^. Measurements were performed three weeks after *in* *vivo* administration of adenovirus carrying si-Dectin-1 (AAV9-si-Dectin-1). AAV9-si-Dectin-1 improved cardiac contractility 21 days after myocardial infarction and inhibited NLRP3 expression, preventing cardiac fibrosis seven days after CAO^[25]^. These data indicate that si-Dectin-1 prevented cardiac remodeling *via* inhibition of cardiac pyroptosis. In another study that the mice underwent permanent CAO^[26]^, myocardial infarction promoted cardiac contractile dysfunction, and left ventricular myocardial fibrosis, and increased the amount of NLRP3, cleaved caspase-1, cleaved IL-1β, cleaved IL-18 in left ventricular tissue for four weeks. Chronic administration of the NLRP3 inhibitor Oridonin (6 mg/kg, intraperitoneal injection) was performed three times a week for two weeks. Oridonin mitigated adverse cardiac remodeling, inhibited pyroptosis and improved the survival rate of mice. In addition, myocardial macrophage and neutrophil influxes were decreased in the Oridonin group^[26]^.

Wild-type and *Camk2n1* gene knockout (*Camk2n1*^−/−^) mice were subjected to permanent CAO in a study^[27]^. Calcium/calmodulin-dependent protein kinase Ⅱ (CaMKⅡδ) inhibitor 1 (Camk2n1) is an endogenous protein inhibitor of CaMKⅡδ^[27]^. Cardiac remodeling was evaluated 28 days after myocardial infarction (MI). As a result, *Camk2n1* gene knockout contributed to an increase in infarct size, the fibrosis area, the collagen Ⅰ, and collagen Ⅲ levels compared with the wild-type mice with MI. Investigators hypothesized that adverse cardiac remodeling could be developed *via* stimulation of the CaMKⅡδ-p38/JNK-NLRP3 pathway, where JNK is c-Jun N-terminal kinase^[27]^. Permanent CAO (10 weeks) triggered cardiac contractile dysfunction in mice^[28]^. Chronic administration of the NLRP3 inhibitor OLT1177 through the chow enriched with OLT1177 (7.50 g/kg) did not mitigate the development of adverse cardiac remodeling^[28]^. The effect of OLT1177 on pyroptosis was not evaluated. Adverse cardiac remodeling was developed six weeks after MI in mice^[29]^.

Chronic administration of traditional Chinese medicine LuQi Formula (LQF) was used to prevent remodeling. LQF is a drug prepared from five medicinal plants. It was dissolved in distilled water to produce the equivalent of the crude drug concentration of 1.78 g/kg and was administered intragastrically for six weeks^[29]^. MI triggered pyroptosis, fibrosis, and hypertrophy of cardiomyocytes, and cardiac contractile dysfunction. LQF ameliorated adverse cardiac remodeling and abolished pyroptosis. Perindopril, a long-acting angiotensin-converting enzyme (ACE) inhibitor, was dissolved in distilled water at a concentration of 0.06 mg/mL. Chronic administration of perindopril mitigated cardiac remodeling and abolished pyroptosis^[29]^. The combined drug LCZ696 (sacubitril/valsartan) was administered at a dose of 68 mg/kg by oral gavage daily for seven days in rats after MI^[30]^. LCZ696 reduced infarct size, mitigated cardiac contractile dysfunction, abolished fibrosis, and decreased the serum IL-1β and IL-18 levels. LCZ696 reduced the amount of NLRP3, caspase-1, IL-1β, IL-18 mRNA and the NLRP and gasdermin D protein level in myocardial tissue. It also reduced the amount of JNK and p-JNK in myocardial tissue. It was hypothesized that LCZ696 mitigated cardiac remodeling, abolished pyroptosis *via* inhibiting the JNK signaling pathway^[30]^.

Chronic administration of angiotensin Ⅱ receptor antagonists (ramipril at a dose of 3 mg/(kg·day) for seven weeks, LCZ696 at a dose of 68 mg/kg orally for four weeks, losartan at a dose of 10 mg/(kg·day) for 10 weeks) could prevent the development of postinfarction cardiac remodeling in rats^[31–33]^. Rats were exposed to H_2_ inhalation with a 2% concentration for 28 days (three hours/day)^[34]^. MCC950 was injected intraperitoneally at a dose of 30 mg/(kg·day) for 28 days^[34]^. Both H_2_ inhalation and MCC950 improved cardiac contractility, reduced the malondialdehyde level, and decreased the amount of collagen Ⅰ, collagen Ⅲ, and α-smooth muscle actin (α-SMA) in left ventricular tissue. In addition, H_2_ inhalation and MCC950 reduced the NLRP3, ASC, cleaved caspase-1, gasdermin D, and IL-1β levels in myocardial tissue. It was proposed that the cardioprotective effect of H_2_ and MCC950 is mediated *via* inhibiting pyroptosis^[34]^.

**Figure 1:**
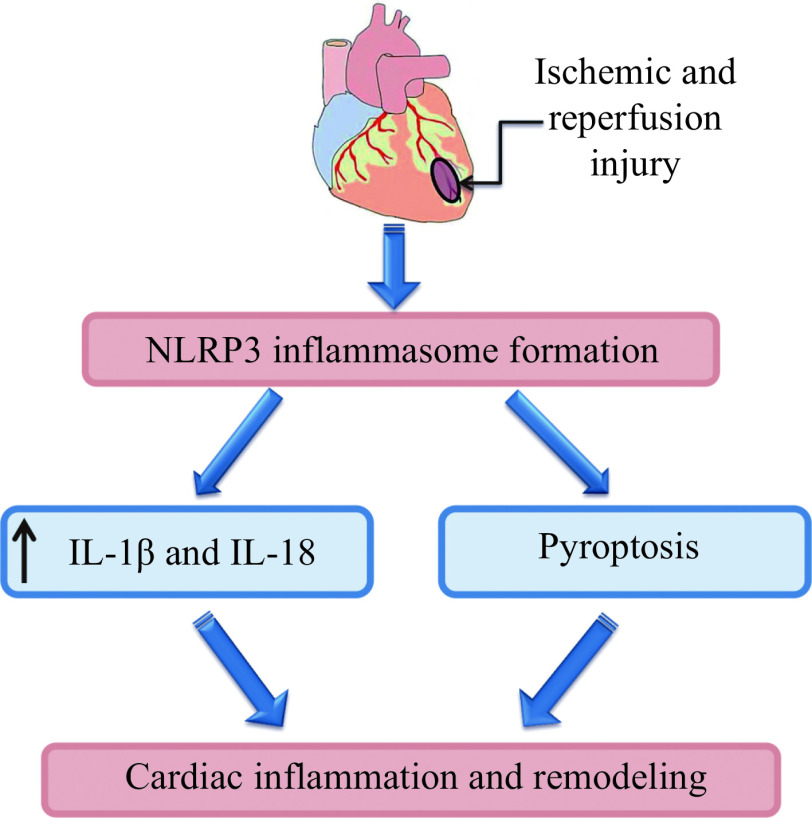
Ischemic and reperfusion injury of the heart causes inflammation and activates pyroptosis that promotes myocardial remodeling.

The aforementioned studies indicate that pyroptosis is involved in postinfarction cardiac remodeling (***Fig. 1***). M1 macrophages promote cardiac fibrosis *via* pyroptosis induction. JNK and CaMKⅡδ are involved in the development of pyroptosis in myocardial tissue. The cathepsin B inhibitor CA-074Me, the NLRP3 inhibitor MCC950, the NLRP3 inhibitor Oridonin, the CaMKⅡδ inhibitor 1 Camk2n1, LQF, perindopril, and valsartan inhibit pyroptosis and prevent postinfarction cardiac remodeling. The NLRP3 inhibitor OLT1177 did not abolish postinfarction cardiac remodeling, possibly because OLT1177 showed no effect on pyroptosis in myocardial tissue.

### The adverse cardiac remodeling in diabetes and a high-fat diet

Streptozotocin-induced diabetes promoted the development of cardiomyocyte pyroptosis^[35]^. This effect peaked eight weeks after streptozotocin injection. Wild-type and db/db diabetic mice underwent permanent CAO^[36]^, and a study was performed two weeks after CAO. Linagliptin, a DPP4 (dipeptidyl-peptidase-4) inhibitor, was mixed with chow (83 mg/kg of chow) and was given for one week before surgery and continued after surgery. As a result, linagliptin improved cardiac contractility, suppressed mRNAs expression of NLRP3, ASC, IL-1β, collagen Ⅰ, and collagen Ⅲ in the border zone of myocardial infarction, and prevented cardiac hypertrophy both in wild-type (WT) and db/db mice^[36]^. Diabetic cardiomyopathy developed in BTBR ob/ob mice (BTBR.Cg-Lepob/WiscJ)^[37]^. Diabetic cardiomyopathy was associated with an increase in the serum glucose, insulin, triglyceride levels, cardiac contractile dysfunction, and fibrosis^[37]^. Diabetes triggered NLRP3, ASC, gasdermin-N, caspase-1, and collagen Ⅰ expression in myocardial tissue, and promoted an increase in the serum TNF-α, IL-1β, IL-6, and IL-18 levels.

Chronic administration of ticagrelor, a P2Y12 receptor antagonist, prevented adverse cardiac remodeling and pyroptosis^[37]^. It was mixed with chow to provide a dose of 100 mg/kg daily for 12 weeks. It was found that ticagrelor increased the phosphorylated-AMP-activated kinase (p-AMPK) level in myocardial tissue. Investigators concluded that anti-pyroptotic and cardioprotective effects of ticagrelor could be mediated *via* activation of AMPK^[37]^. Alloxan-induced diabetes triggered cardiac hypertrophy, atrial hypertrophy, and cardiac contractile dysfunction, increased atrial fibrillation inducibility and atrial interstitial fibrosis in rabbits^[38]^. Diabetes resulted in an increase in NLRP3 content in atrial tissue, a rise in the serum IL-1β and IL-18 concentrations. Investigators proposed that diabetes mellitus can induce atrial fibrillation and remodeling *via* stimulation of pyroptosis^[38]^. It was found that streptozotocin-induced type 1 diabetes resulted in cardiac contractile dysfunction, and an increase in toll-like receptor-4 (TLR4), NLRP3, caspase-1, IL-1β, IL-6, and TNF-α expression in myocardial tissue in mice^[39]^.

Streptozotocin triggered inflammatory dendritic cell invasion, cardiomyocyte hypertrophy, and interstitial fibrosis. Bone morphogenetic protein-7 (BMP-7) was administered with 200 mg/kg intraperitoneally. BMP-7 inhibited inflammation, pyroptosis, and improved cardiac contractility^[39]^. MCC950 was injected at a dose of 5 mg/kg intraperitoneally twice per week for six weeks after MI of mice with streptozotocin-induced diabetes^[40]^. Diabetes aggravated adverse cardiac remodeling, increased the mortality rate, cardiac hypertrophy, the fibrotic area, and enhanced IL-1β, IL-6, TNF-α, and IL-18 expression in myocardial tissue. It also enhanced NLRP3 and caspase-1 expression. MCC950 improved cardiac contractility, reduced cardiac hypertrophy and the fibrotic area, inhibited NLRP3 and caspase-1 expression, and suppressed inflammation in mice with MI and diabetes^[40]^. It was suggested that diabetes aggravates cardiac remodeling after MI through stimulation of pyroptosis^[40]^.

It has been demonstrated that a high-fat diet (HFD) which mimics metabolic syndrome and type 2 diabetes in rats promotes an increase in the NLRP3, caspase-1, ASC, and IL-1β level in the heart^[41–42]^. Aminooxyacetic acid (AOAA), an inhibitor of aspartate aminotransferase, was found to have effects of decreasing infarct size in mice with permanent CAO and increasing left ventricle ejection fraction 28 days after myocardial infarction^[43]^. AOAA was administrated at a dose of 10 mg/kg intraperitoneally daily. It inhibited NLRP3, IL-1β, and caspase-1 expression in myocardial tissue three days after myocardial infarction. The authors concluded that AOAA attenuated postinfarction cardiac remodeling *via* inhibition of the NLRP3-caspase1/IL-1β pathway^[43]^. WT, *Nlrp3*^−/−^, and *ASC*^−/−^ male mice were used for exposure to a high-fat diet (HFD; 60 cal% fat) or a control diet for 52 weeks^[44]^. HFD triggered obesity, insulin resistance, hepatic steatosis, cardiac hypertrophy, and contractile dysfunction. NLRP3 or ASC knockout abolished obesity, insulin resistance, hepatic steatosis, and contractile dysfunction but did not prevent cardiac hypertrophy. Rats were divided into three groups in another study: (1) a control diet; (2) HDF for 12 weeks; (3) HFD + exercise^[45]^. The rats in the exercise groups were trained on a motor treadmill seven days/week for 12 weeks. HFD induced obesity, and an increase in blood lipid level, and levels of P2X7, NLRP3, interleukin-1β, caspase-1, and collagen Ⅰ in myocardial tissue. Exercising training eliminated these alterations^[45]^. Palmitic acid resulted in pyroptosis in H9C2 cells. The P2X7 receptor inhibitor A438079 prevented pyroptosis in H9C2 cells^[45]^.

Mice were fed a HFD for eight weeks before injection of streptozotocin and were maintained for another eight weeks^[46]^. Diabetes induced cardiomyocyte hypertrophy, cardiac hypertrophy, interstitial fibrosis, oxidative stress, and cardiac contractile dysfunction, and increased gasdermin-D-N, and IL-18 expression in myocardial tissue^[46]^. Diabetes and a HFD could be a trigger of adverse cardiac remodeling. Pyroptosis is involved in HFD-induced and diabetes-induced cardiac remodeling. Linagliptin, a DPP4 inhibitor, ticagrelor, a P2Y12 receptor antagonist, bone morphogenetic protein-7, MCC950 improved cardiac contractility and inhibited pyroptosis in animals with diabetic cardiomyopathy. Aminooxyacetic acid and the P2X7 receptor inhibitor A438079 prevented the development of heart failure and pyroptosis in HFD-induced cardiomyopathy.

### The role of pyroptosis in angiotensin-Ⅱ and pressure overload-induced cardiac remodeling

Serum-glucocorticoid regulated kinase 1 (SGK1) is involved in angiotensin-Ⅱ induced myocardial fibrosis^[47]^. It has been found that the selective SGK1 inhibitor EMD638683 prevents angiotensin-Ⅱ-induced cardiac fibrosis in mice *via* inhibition of NLRP3 and IL-1β expression in myocardial tissue^[48]^. Mice were subjected to transverse aortic constriction (TAC)^[23]^. MCC950 was administered at a dose of 5 mg/(kg·day) through subcutaneous osmotic pumps for four weeks. TAC triggered cardiac fibrosis and heart failure, while MCC950 improved cardiac contractility and abolished cardiac fibrosis^[23]^. TAC promoted the development of heart failure, and cardiac fibrosis and increased the NLRP3, gasdermin D, ASC, cleaved caspase-1, p-NFκB, IL-1β, and IL-18 levels in myocardial tissue in mice^[49]^. Mice were given URMC-099 (a mixed lineage kinase type 3 inhibitor) at a dose of 10 mg/kg intraperitoneally, traditional Chinese medicine Xinyang tablet at a dose of 1.36 g/kg by gavage, and perindopril at a dose of 0.607 mg/kg by gavage seven days before TAC^[49]^. All drugs abolished heart failure, cardiac fibrosis and pyroptosis^[49]^. Ma *et* *al* confirmed that TAC administration for eight weeks induced heart failure, cardiac fibrosis, oxidative stress, inflammation and pyroptosis in mice^[50]^. TAC (administration for six weeks) triggered heart failure, cardiac hypertrophy, and cardiomyocyte hypertrophy in mice^[51]^. In addition, it increased the amounts of p-p38, p-JNK, p- CaMKⅡδ, phosphorylated extracellular signal-regulated kinase (p-ERK), and ASC in myocardial tissue. It also increased the pro-inflammatory cytokine (IL-1β, IL-6, and TNF-α) contents^[51]^, and promoted macrophage infiltration, including both M1 and M2 macrophages. MCC950 was administered at a dose of 10 mg/kg intraperitoneally once per day at two weeks after TAC surgery for four weeks. It improved cardiac contractility, prevented cardiac fibrosis and activation of enzymes, inhibited cytokine production, and suppressed macrophage infiltration. The role of p-p38, p-JNK, p-CaMKⅡδ, p-ERK in TAC-induced pyroptosis remains unclear.

It was demonstrated that angiotensin-Ⅱ induced myocardial fibrosis and the activation of the NLRP3 inflammasome is mediated *via* stimulation of calcium/calmodulin-dependent protein kinase Ⅱ δ (CaMKⅡδ) and NF-κB activation in mice^[52]^. It was proposed that the NLRP3 inflammasome can activate CaMKⅡδ in atrial tissue of patients with atrial fibrillation^[53]^. The involvement of CaMKⅡδ and NF-κB in TAC-induced cardiac fibrosis in mice is confirmed by data of other investigators^[54]^. In addition, deletion of CaMKⅡδ in cardiomyocytes attenuates TAC-induced accumulation of CD68^+^ macrophages in cardiac tissue. The same investigators obtained evidence that intracardiac catheter delivery of cathepsin G caused the non-canonical pyroptosis pathway with IL-1β and IL-18 involvement^[54]^. Mice were subjected to aortic banding (AB) for eight weeks^[55]^. Aortic banding triggered the development of heart failure, cardiac hypertrophy, cardiomyocyte hypertrophy, and cardiac fibrosis, and increased NLRP3, NFκB, ASC, IL-1β, cleaved caspase-1 expression, and inflammatory cell infiltration^[55]^. TAC (administration for eight weeks) induced heart failure, and cardiac fibrosis^[56]^. Gasdermin D, cleaved caspase-1, IL-1β, IL-18, NLP3, and p-NFκB expression in myocardial tissue peaked after one week^[56]^. The expression of these proteins decreased eight weeks after TAC but remained above normal. TAC (administration for four weeks) promoted the development of heart failure, cardiac hypertrophy, and an increase in P2X7 receptor, NLRP3, and IL-1β expression in myocardial tissue^[57]^. The P2X7 receptor antagonist brilliant blue G (BBG) was injected at a dose of 30 mg/kg intraperitoneally three times per week. BBG ameliorated heart failure and inhibited pyroptosis. Spontaneously hypertensive rats were fed an HFD for 10 weeks^[58]^. This combined effect contributed to cardiac hypertrophy and cardiac fibrosis.

Valsartan, an angiotensin Ⅱ receptor antagonist, was given 30 mg/(kg·day) intragastrically for 10 weeks. Decoction of Chinese medicine Huoxue Qianyang Qutan Recipe (HQQR) was given for 10 weeks. Both valsartan and HQQR prevented the development of cardiac hypertrophy and cardiac fibrosis. Isolated cardiac fibroblasts (CFs) were incubated with angiotensin Ⅱ (1 µmol/L) for 48 hours^[58]^. As a result, angiotensin Ⅱ increased IL-1β, NLRP3, and caspase-1 expression in CFs. Valsartan inhibited pyroptosis of CFs. HQQR exhibited the same effect. Consequently, angiotensin Ⅱ plays an important role in pressure overload-induced cardiac remodeling. Angiotensin Ⅱ [1500 ng/(kg·min)] was infused for seven days by osmotic mini-pumps which were implanted subcutaneously^[59]^. Angiotensin Ⅱ induced cardiac hypertrophy, cardiomyocyte hypertrophy, and cardiac fibrosis, but did not result in heart failure in mice. It increased the expression of NLRP3, pro-caspase-1, cleaved caspase-1, pro-IL-1β, and IL-1β in myocardial tissue, and contributed to macrophage invasion in the myocardium. Darapladib, a lipoprotein-associated phospholipase A2 (Lp-PLA2) inhibitor, was administered by gavage (50 mg/kg) once per day for three days before Angiotensin Ⅱ infusion. It abolished angiotensin Ⅱ-triggered cardiac hypertrophy, cardiomyocyte hypertrophy, and cardiac fibrosis, and inhibited pyroptosis and macrophage invasion. It was concluded that darapladib exhibited anti-fibrotic, anti-inflammatory, and anti-pyroptotic effects.

We reported above that pyroptosis is involved in pressure overload-induced cardiac remodeling. However, *NLRP3*^−/−^ homozygous mutation accelerated cardiac hypertrophy, fibrosis, and myocardial inflammation in pressure overload-induced cardiac remodeling in mice^[60]^. It was found that TLR4 expression was up-regulated in NLRP3-deficient mice hearts. It was suggested that NLRP3 deficiency contributed to pressure overload-induced cardiac remodeling *via* an increase in TLR4 expression^[60]^.

These data indicate that pyroptosis is involved in angiotensin-Ⅱ and pressure overload- induced adverse cardiac remodeling. The SGK1 inhibitor EMD638683, the P2X7 receptor inhibitor brilliant blue G, HQQR, valsartan, URMC-099, Xinyang Tablet, and perindopril can prevent TAC-induced cardiac remodeling and pyroptosis in myocardial tissue.

## Pyroptosis in acute myocardial infarction

Information on the involvement of pyroptosis in I/R injury of the human heart is limited. It was found that the plasma IL-18 level in patients with STEMI was elevated by 2.4 fold compared with the control subjects^[61]^. A positive correlation was demonstrated between IL-18 and cardiac troponin-I. The plasma IL-18 level on admission might predict a 60-day adverse clinical outcome^[61]^. Patients with acute coronary syndrome (ACS) showed 2-fold higher of serum IL-18 level comparing with the control subjects^[62]^. Long-term, cardiovascular mortality was related to an increase in the serum IL-18 concentration in patients with ACS^[63]^. The plasma IL-18 level was increased for 3.9 fold in AMI patients compared with that of the stable angina pectoris patients^[64]^. According to Xie *et* *al*^[65]^, the serum IL-18 level was 2-fold higher in patients with AMI than in healthy volunteers. In ACS patients, the plasma IL-18 concentrations elevated after the occurance of acute events and remained increased for six months^[66]^.

In 2015, a paradoxical result was obtained that the plasma IL-18 level in patients with STEMI 12 hours after percutaneous coronary intervention (PCI) by 49% lower than that in healthy volunteers^[67]^. The IL-18 level remained low for at least 72 hours after PCI. The authors did not provide a convincing explanation^[67]^. Some investigators argue that monocytes are a major source of serum IL-18^[65]^.The plasma IL-1β level was elevated for about two fold in patients with AMI compared with the healthy volunteer^[68]^. It was found that the increased caspase-1 and interleukin-1β levels were associated with left ventricular hypertrophy and cardiac remodeling in patients with STEMI and PCI^[69]^. Therefore, it is unclear whether an increase in the IL-18 concentration is a result of pyroptosis of cardiomyocytes or a consequence of monocytes activation.

Coronary bypass surgery is accompanied by cardioplegic arrest, which is essentially global ischemia of the heart. Investigators found that the *NLRP3* mRNA level was decreased in tissue from the right atrium of cardiac surgery patients compared with the controls^[70]^. This result is inconsistent with our understanding of the activation of pyroptosis in myocardial tissue after I/R. Thus, at the present time, there is no convincing evidence for the activation of pyroptosis in the human heart, because IL-1β and IL-18 can be synthesized not only in the myocardium but can also enter the bloodstream from other organs and tissues, for example, the leukocytes.

## The regulation of pyroptosis in the heart

The regulation and inhibition of pyroptosis is an important problem of cardiology and pharmacology. In 2011, it was demonstrated that the activation of the purinergic P2X7 receptor might be a trigger of pyroptosis in the border zone of the murine heart after permanent CAO, because both P2X7 siRNA and the purinergic P2X7 receptor inhibitor PPADS abolished pyroptosis^[20]^. In 2017, the evidence was obtained that eNOS was involved in a serelaxin-induced decrease in infarct size and inhibition of caspase-1^[71]^. NF-κB plays an important role in the activation of pyroptosis. Inhibition of NF-κB with pyrrolidine dithiocarbamate (PDTC) suppressed pyroptosis of H9C2 cells induced by oxygen-glucose deprivation (OGD)^[72]^. A non-selective reactive oxygen species (ROS) scavenger N-acetyl-cysteine also inhibited OGD-induced pyroptosis^[72]^. β-adrenergic receptors (β-ARs) also play an important role in the regulation of pyroptosis. In 2018, it was demonstrated that the β-AR agonist isoproterenol induced cardiac injury, the activation of oxidative stress, and enhancement of IL-1β, IL-18, NLRP3 and ASC production in the myocardium^[73]^. Isoproterenol at a dose of 5 mg/kg induced an increase of the IL-1β, IL-18, and caspase-1 levels in the myocardium. In both cases, a cardiotoxic effect of isoproterenol had been shown, since its intravenous injection at a dose of 2 µg/kg is sufficient for the chronotropic response to isoproterenol^[74]^, thereby further studies are required on the role of catecholamines and β-ARs in the regulation of pyroptosis.

In a study performed on mice with permanent CAO, the selective cannabinoid receptor-2 (CB2) agonists JWH-133 (10 mg/kg) decreased the IS/AAR ratio by about 33% and improved cardiac contractility 6 hours after myocardial infarction^[75]^. The cardioprotective effect was associated with reduced serum IL-1β, IL-18 levels and decreased IL-1β, IL-18, NLRP3, and caspase-1 expression in the myocardium. Investigators reported that the selective CB2 receptor antagonist AM630 abolished the cardioprotective effect of JWH-133. These data show that the activation of CB2 receptor can increase cardiac tolerance to I/R *via* inhibition of pyroptosis.

There is indirect evidence that kinases may be involved in the regulation of pyroptosis. A study was performed on wild-type rats and PTEN knockout rats^[76]^. It is reported that phosphate and tension homolog deleted on chromosome ten (PTEN) is phosphatase which dephosphorylates phosphatidylinositol-3,4,5-trisphosphate, a potent activator of 3-phosphoinositide-dependent kinase (PDK) and Akt kinase^[77]^. After PTEN knockout, cardiac tolerance to I/R was enhanced and the NLRP3, caspase-1, and IL-1β protein levels in the myocardium were decreased^[76]^. This result can be considered as indirect evidence of the involvement of Akt and PTEN in the regulation of pyroptosis of cardiomyocytes during I/R.

The pyruvate dehydrogenase (PDH) and Sirtuin-1, a member of the NAD-dependent Sirtuin family of histone/protein deacetylases, were shown to be involved in the regulation of pyroptosis of cardiomyocytes^[78]^. Mice were subjected to CAO (45 minutes) and reperfusion (6 hours)^[78]^. The sirtuin-1 agonist SRT1720 improved cardiac contractility during cardiac reperfusion in wild-type mice, decreased *NLRP3* mRNA expression in the heart after I/R, reduced the serum IL-1β and IL-18 concentration, and inhibited mitochondrial ROS production^[78]^. Inducible cardiomyocyte-specific PDH E1α knockout promoted a reduction in the serum IL-1β and IL-18 levels after I/R and decreased NLRP3 and p-PTEN contents in myocardial tissue^[78]^. These findings show that PDH and Sirtuin-1 may be endogenous regulators of pyroptosis.

Periostin is a secreted extracellular matrix protein. It was found that hypoxia/reoxygenation (H/R) up-regulated mRNA expression of ASC, NLRP3, gasdermin D, caspase-1, IL-1β, and IL-18 in H9C2 cells^[79]^. Periostin siRNA inhibited pyroptosis marker expression. This result indicates that periostin may be one of endogenous regulators of pyroptosis.

Small endogenous non-coding RNAs (microRNA) are involved in myocardial I/R injury^[80]^. In recent years, evidence has emerged that microRNAs regulate pyroptosis of cardiomyocytes during H/R. It was found that I/R of the heart and H/R of H9C2 cells caused miR-424 expression^[81]^. The miR-424 inhibitor was studied to determine its role in pyroptosis. It was found that the miR-424 inhibitor suppressed caspase-1, IL-1β, and IL-18 expression. I/R up-regulated miR-29a expression in myocardial tissue in mice^[82]^. The miRNA-29a inhibitor up-regulated Sirtuin-1 expression and down-regulated NLRP3, caspase-1, and IL-1β expression in H9C2 cardiomyocytes subjected to H/R^[82]^. H/R of cardiomyocytes up-regulated miR-149 expression and elevated NLRP3, caspase-1, IL-1β, and IL-18 production by H9C2 cardiomyocytes after H/R^[83]^. The miR-149 inhibited pyroptosis marker expression. The miR-132 level significantly elevated the heart of mice after I/R^[84]^. H/R caused the activation of pyroptosis in H9C2 cells subjected to H/R^[84]^.

The role of peroxisome proliferator-activated receptor-gamma coactivator (PGC)-1α/nuclear factor erythroid-2-related factor 2 (Nrf2) signaling was studied in the effects of miR-132. miR-132 inhibitor activated PGC-1α/Nrf2 signaling and decreased the malonyl dialdehyde level and down-regulated NLRP3, caspase-1 and IL-1β expression in H9C2 cells. It was established that M2 macrophage-derived exosomes (M2-exos) containing miR-148a increased the survival rate of neonatal rat cardiomyocytes under the condition of H/R^[85]^. M2-exos (2–3 μg per rat) were injected two hours before CAO (30 minutes) and reperfusion (two hours) in rats. They decreased the IS/AAR ratio approximately by 60%. M2-antagomiR-148a abolished the protective effect of M2-exos both *in* *vivo* and *in* *vitro*. Investigators linked the cardioprotective effect of M2-exos and miR-148a to inhibition of the TLR4/NF-κB/NLRP3 inflammasome signaling pathway^[85]^. Recently, it has been demonstrated that H/R induced an increase in pyroptosis marker expression and a decrease in miR-703 content in isolated murine cardiomyocytes^[86]^. miR-703 mimics increased cell viability and down-regulated pyroptosis marker expression in cardiomyocytes subjected to H/R. The miR-703 inhibitor decreased cell viability and up-regulated the mRNA level of NLRP3, IL-1β, IL-18, and caspase-1 in cardiomyocytes in H/R.

**Figure 2:**
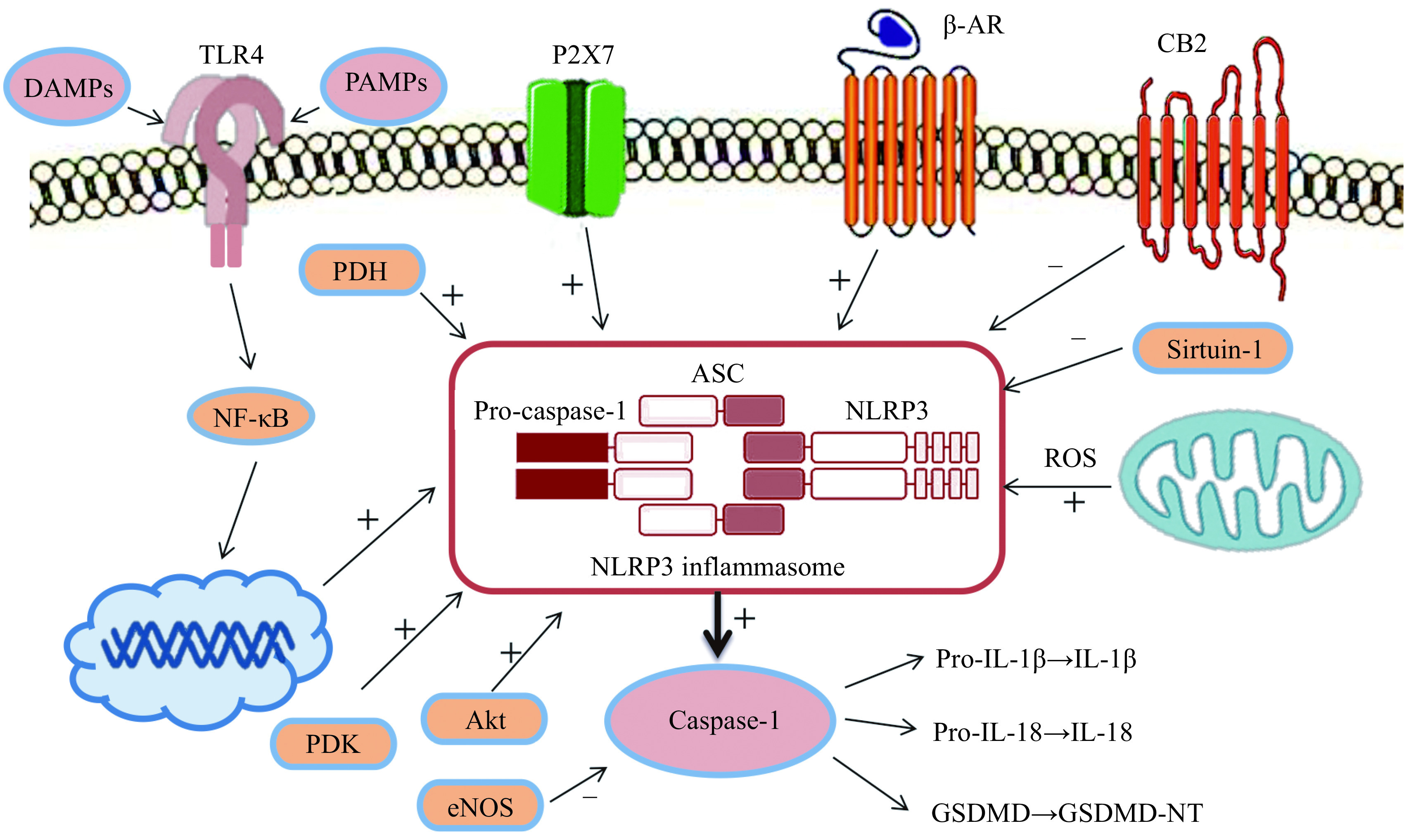
The regulation of pyroptosis of the heart.

The aforementioned studies present convincing evidence that microRNAs can regulate pyroptosis of cardiomyocytes. It was found that NF-κB, ROS, CB2 receptor, PTEN, Sirtuin-1, and periostin could be involved in the regulation of pyroptosis of cardiomyocytes (***Fig. 2***). In general, information on the regulation of pyroptosis of cardiomyocytes is fragmentary and incomplete. It is unknown what is the role of endogenous opioids, cannabinoids, adenosine, bradykinin, glucagon-like peptide, IL-6, epoxyeicosatrienoic acids, sphingosine-1-phosphate, fibroblast growth factor, urocortin, erythropoietin, and natriuretic peptides in the regulation of pyroptosis of cardiomyocytes. They increased cardiac tolerance to I/R^[87–89]^. The effect of ischemic preconditioning, postconditioning, remote conditioning on pyroptosis in the myocardium also remains unknown.

## Pyroptosis and inflammation

Pyroptosis is inflammation-related programmed cell death. It triggered the release of inflammatory cytokines IL-1β and IL-18 through the pores formed in the plasma membrane by gasdermin^[90]^. These cytokines can induce inflammation. Pyroptotic cell death causes DAMPs released from dead cells^[91]^. DAMPs can also induce inflammation by activation of TLRs^[91–92]^. DAMPs can cause inflammation and the development of inflammatory diseases, for example, *via* TLRs, its receptor for advanced glycation end-products RAGE (receptor for advanced glycation end products), to activate NF-κB and receptors involved in inflammation^[93]^. Long-term pyroptosis may promote the development of chronic inflammation and fibrosis^[94]^. Zhang *et* *al* suggest that pyroptosis-triggered inflammation causes cardiac fibrosis and adverse cardiac remodeling^[94]^. Song *et* *al* have a similar opinion^[95]^. Consequently, pyroptosis can induce acute and chronic inflammation, cardiac fibrosis and adverse remodeling of the heart (***Fig. 1***).

## The crosstalk between pyroptosis and other forms of programmed cell death

### The crosstalk between pyroptosis and apoptosis

Rats were subjected to CAO (30 minutes) and reperfusion (120 minutes)^[96]^. Ischemia/reperfusion induced infarct size, an increase in the 4-hydroxynonenal, NLRP3, and cleaved caspase levels, and an increased number of TUNEL positive (*i.e.*, apoptotic) cells. Streptozotocin-induced diabetes aggravated I/R cardiac injury, increased the 4-hydroxynonenal, NLRP3, cleaved caspase levels, and the number of apoptotic cells^[96]^. The NADPH oxidase 2 (Nox-2) inhibitor Vas2870 (2 mg/kg) was administered intravenously before I/R. Vas2870 reduced infarct size, inhibited oxidative stress, decreased the number of apoptotic cells, and reduced NLRP3 expression in myocardial tissue. Consequently, apoptosis and pyroptosis change synchronously. Streptozotocin-induced diabetes triggered both pyroptotic and apoptotic cardiomyocyte death^[35]^. Administration of *NLRP3*-miRNA inhibited both pyroptosis and apoptosis^[35]^. These data indicate that pyroptosis and apoptosis develop synchronously. Streptozotocin-induced diabetes triggered both pyroptosis and apoptosis in the rat heart^[97]^. Palmitic acid triggered pyroptosis and apoptosis of H9c2 cells^[45]^. The P2X7 receptor inhibitor A438079 mitigated both pyroptosis and apoptosis^[45]^. Diabetes triggered NLRP3, ASC, gasdermin-N, caspase-1, and collagen Ⅰ expression and increased the number of TUNEL positive cells in myocardial tissue of ob/ob mice^[37]^. Dapagliflozin, a sodium-glucose-cotransporter-2 inhibitor, suppressed pyroptosis and apoptosis of cardiomyocytes^[37]^. Aortic banding for eight weeks promoted pyroptosis and apoptosis of cardiomyocytes in mice^[55]^.

In a study, mice were subjected to CAO (30 minutes) and reperfusion (120 minutes)^[98]^. I/R triggered both pyroptosis and apoptosis. The hyperuricemia was induced by intraperitoneal injection of a potassium oxonate suspension dissolved in 0.5% sodium carboxymethyl cellulose at 300 mg/(kg·day) for 14 consecutive days. Hyperuricemia aggravated I/R cardiac injury and enhanced pyroptosis and apoptosis. The high glucose concentration promoted apoptotic and pyroptotic death of H9C2 cells^[99]^. Doxorubicin (2 µmol/L, 48 hours) stimulated both pyroptosis and apoptosis of H9C2 cells^[100]^. Streptozotocin-induced diabetes promoted adverse cardiac remodeling, pyroptosis and apoptosis in mice^[40]^. The role of transcription factor interferon regulatory factor 2 (IRF2) was studied in the regulation of pyroptosis in mice with MI^[101]^. Lentivirus-irF2-short hairpin (sh) RNA (sh-IRF2 forward, GGTCCTGACTTCAACTATA) was injected. Myocardial infarction increased IRF2 expression and stimulated both pyroptosis and apoptosis in myocardial tissue. Sh-IRF2 lentivirus prevented pyroptotic and apoptotic cardiomyocyte death.

These data indicate that the pyroptosis and the apoptosis pathways alter synchronously in the heart. Consequently, it could be hypothesized that there is a crosstalk between pyroptosis and apoptosis.

### Pyroptosis and autophagy

Primary cardiomyocytes isolated from neonatal C57BL/6 mice were incubated with the high glucose (HG) concentration for 24 hours^[102]^. HG triggered an increase in the NLRP3, caspase-1, gasdermin-D-N, and IL-1β levels and reduced phosphorylated AMP-activated protein kinase (p-AMPK), and microtubule-associated protein 1 light chain 3 (LC3-Ⅱ protein). HG induced the expression of mammalian target of rapamycin (mTOR) which is an inhibitor of autophagy. Metformin, an AMPK activator, reduced the NLRP3, caspase-1, gasdermin-D-N, mTOR, and IL-1β levels and increased the amount of LC3-Ⅱ. The AMPK inhibitor compound C abolished all effects of metformin^[102]^. Consequently, pyroptosis and autophagy were altered in the opposite way in cardiomyocytes. Primary cardiomyocytes were isolated from the myocardium of neonatal C57BL/6 mice^[103]^. Cardiomyocytes were incubated with lipopolysaccharide (LPS) to mimic sepsis-induced injury of isolated cells. LPS contributed to an increase in the amount of NLRP3, gasdermin-D-N, cleaved IL-1β, caspase-1, and p-mTOR. In contrast, the p-AMPK, LC3-Ⅱ and Beclin levels were reduced. P62 content was increased^[103]^. P62 protein (sequestosome 1/SQSTM1) plays a key role in mitophagy^[104]^.

Hypoxia (two hours)/reoxygenation (two hours) induced the expression of gasdermin D and IL-1β in wild-type human cardiac microvascular endothelial cells (HCMECs)^[105]^. Transfection of these cells with Beclin1 lentivirus triggered Beclin 1 over-expression and prevented H/R-induced pyroptosis of HCMECs^[105]^. CAO (45 minutes) and reperfusion were performed in WT mice and transgenic mice with Beclin 1 over-expression. Beclin1 over-expression reduced infarct size, decreased the no-reflow area, and attenuated pyroptosis^[105]^. Consequently, activation of autophagy switches the pyroptotic pathway of cell death on autophagy. There is evidence that autophagy could demonstrate positive effects, for example, mitophagy could attenuate inflammation-mediated myocardial injury, and increase cardiac resistance to ischemic and reperfusion injury^[106–109]^. Human stem cell-derived cardiomyocytes (hiPSC-CMs) were incubated with LPS^[109]^. LPS induced cell death, and lactate dehydrogenase (LDH) release, increased the NLRP3, cleaved caspase-1, gasdermin-D-N, cleaved IL-1β, and cleaved IL-18 levels^[110]^. LPS reduced the amount of LC3-Ⅱ, and lysosomal-associated membrane protein 1 (LAMP1) and increased the p62 level. These data indicate that LPS stimulates pyroptosis, inhibits autophagy but activates mitophagy. Melatonin (10 µmol/L) increased cell viability, reduced LDH release, inhibited pyroptosis, and stimulated autophagy, but reduced the p62 level^[110]^. Consequently, a pyroptosis process and autophagy process change in exactly the opposite way. Mice were fed a HFD for eight weeks prior to injection of streptozotocin and were maintained for another eight weeks^[46]^. Diabetes promoted the decrease of LC3-Ⅱ content, increased the p62, NLRP3, gasdermin-D-N, and IL-18 levels in myocardial tissue. These data indicate that diabetes contributes to inhibiting autophagy and activating pyroptosis.

Consequently, autophagy is reciprocally related to pyroptosis processes. The molecular mechanism of interactions of pyroptosis and autophagy in the heart was unclear before. It was hypothesized that autophagy could negatively regulate pyroptosis, inhibit inflammatory cytokine release through elimination of DAMPs, PAMPs, inflammasomes, and block other proteins involved in pyroptosis^[111]^. Other investigators hypothesized that histone deacetylase-6 was involved in the crosstalk between autophagy and pyroptosis^[112]^.

## Pharmacological approaches to prevention of pyroptosis of the heart

The aforementioned studies indicate that it is possible to achieve an increase in cardiac tolerance to ischemia or reperfusion and to prevent adverse cardiac remodeling, diabetic cardiomyopathy, and metabolic syndrome-triggered cardiomyopathy by inhibiting pyroptosis of cardiomyocytes and endothelial cells. Effective pyroptosis inhibition can be achieved by blockade of TLR4 expression, NLRP3 inflammasome inhibition, and inhibition of caspase-1. It could be hypothesized that an effective approach to prevention of pyroptosis of cardiomyocytes will include blockade of IL-1β and IL-18 receptors.

Artificial exosomes containing microRNA have been reported to be useful in clinical practice. It is possible that microRNA inhibiting the expression of NLRP3, cleaved caspase-1, cleaved IL-1β, and IL-18 will soon be used for treating AMI and preventing adverse cardiac remodeling. The traditional Chinese herbal medicines (HQQR, LQF, and Xinyang Tablet) are noteworthy. The main disadvantage of these drugs is the inability of parenteral administration and the high likelihood of allergic reactions since they contain many plant components. ACE inhibitors and angiotensin Ⅱ receptor blockers can also inhibit pyroptosis and prevent adverse cardiac remodeling. Many pharmacological agents (metformin, scutellarin, ticagrelor, aminooxyacetic acid, the cathepsin B inhibitor CA-074Me, darapladib, Nox-2 inhibitors, linagliptin, luteolin, bone morphogenetic protein-7, P2X7 receptor antagonists, emodin, kanglexin, serelaxin, β-asarone, serelaxin, and 9,20-epoxydocosapentaenoic acid) inhibit pyroptosis. However, mechanisms of their cardioprotective effect and pyroptosis inhibition remain to be clarified.

## Conclusions

Pyroptosis is involved in ischemic and reperfusion injury of the heart^[17]^. The aforementioned studies indicate that inhibition of pyroptosis could prove to be an effective approach to prevent postinfarction and pressure overload-induced adverse cardiac remodeling. The NLRP3 inhibitors prevent the development of diabetic cardiomyopathy. Diabetes is not a major barrier to inhibit pyroptosis and postinfarction cardiac remodeling. HFD promotes activation of pyroptosis in the heart. Therefore, future studies should focus on developing a more thorough understanding of the mechanisms of the protective effect of pyroptosis inhibitors which could eventually lead to the creation of one or more of these inhibitors in clinical practice. It was demonstrated that autophagy stimulation promotes inhibition of pyroptosis. The activation of pyroptosis promotes apoptosis stimulation. The molecular mechanisms of interactions of pyroptosis, apoptosis, autophagy in I/R of the heart or cardiac remodeling remain unknown. It is unclear whether pyroptosis interacts with ferroptosis or necroptosis in I/R of the heart or remodeling. It should be emphasized that most of the current knowledge is based on animal models and that clinical data are limited. Translation of experimental data to clinical practice is difficult because animals usually do not have comorbid pathology.
